# Fracture Toughness and Blocking Force of Temperature-Sensitive PolyNIPAAm and Alginate Hybrid Gels

**DOI:** 10.3390/gels8050324

**Published:** 2022-05-23

**Authors:** Yong-Woo Kim, Do Yoon Kim, Jeong-Yun Sun

**Affiliations:** 1 Department of Materials Science and Engineering, Seoul National University, Seoul 151-742, Korea; ywkim0710@snu.ac.kr (Y.-W.K.); dykim0423@snu.ac.kr (D.Y.K.); 2 Research Institute of Advanced Materials, Seoul National University, Seoul 151-742, Korea

**Keywords:** tough hydrogels, actuators, thermo-sensitive materials, alginates, poly(N-isopropylacrylamide)

## Abstract

In the field of actuator materials, hydrogels that undergo large volume changes in response to external stimuli have been developed for a variety of promising applications. However, most conventional hydrogels are brittle and therefore rupture when they are stretched to moderate strains (~50%). Thus, gels to be used for actuators still require improved mechanical properties and actuation performance. In this study, we synthesized a tough and thermo-sensitive hydrogel with a large actuation force by forming interpenetrating networks between covalently crosslinked poly(N-isopropylacrylamide) and ionically crosslinked alginate. Poly(N-isopropylacrylamide) was used as a thermo-sensitive actuation material, and alginate was found to enhance the mechanical properties of the hydrogels. Due to the enhanced elastic modulus and energy dissipation in the hybrid gel, the toughness was increased by a factor of 60 over that of pure PNIPAAm gel. Further, based on the results showing that the hybrid gel exhibits an actuation force that is seven times higher than that of pure PNIPAAm gel, the hybrid gel is more applicable to real actuators.

## 1. Introduction

Hydrogels are crosslinked network polymers with such high hydrophilic moiety that they contain large amounts of water, occupying more than 90 wt. % of the hydrogel. Some hydrogels—including polyelectrolytes, poly(N-isopropylacrylamide), azo compound polymers, antigen/antibody-grafted polymers, and Belousov–Zhabotinsky (BZ) gels—undergo large volume changes with varying water concentration, as they respond to various stimuli such as Ph [1], temperature [2,3], light [4], antigen [5], and periodic changes in solubility by Ru(bpy)_3_ [6,7]. Due to their large volumetric change, some stimulus-responsive hydrogels have been studied as actuator materials [8,9,10,11]. However, the mechanical behaviors of hydrogels restrict their applicability, which is limited by their mechanical behaviors. To elaborate, the previously reported stimulus-responsive hydrogels are unsuitable for actuators against gravity because they exhibit brittle properties and a low fracture toughness of approximately 20 J m^−2^.

Various types of hydrogels, including fiber-reinforced gel [12] and silica nanoparticle-grafted hydrogel [13], have been developed in attempts to enhance the fracture toughness. One study achieved a remarkable enhancement of fracture energy greater than 1000 J m^−2^ by forming a double-network structure [14]. Double networks contain interpenetrated networks between covalently crosslinked short and long chains. In another work, Sun and colleagues reported the synthesis of hydrogels from polymers to form ionically and covalently crosslinked networks, and they attributed the resulting toughness of the gel to the synergy of two mechanisms: crack bridging by the network of covalent crosslinks and energy dissipation by unzipping the network of ionic crosslinks [15]. In that study, an extreme fracture toughness of approximately 9000 J m^−2^ was achieved by introducing reversible ionic crosslinks of alginate into covalent crosslinks of polyacrylamide instead of interpenetrating two strong, covalently crosslinked networks [15]. They also reported highly stiff and tough hydrogels by fully utilizing ionic crosslinking sites of alginate chains [16]. However, although these tough gels exhibited excellent mechanical properties, they were not stimulus responsive.

In a recent study, a topological hydrogel with sliding-ring crosslinkers was synthesized to enhance the mechanical properties of actuating materials [17]. This hydrogel exhibited high stretchability, which was achieved by using polyrotaxane sliding rings that can move along the chain. It also demonstrated thermo-sensitive properties and high stretchability. Stretchability is an important issue for high fracture toughness, as it facilitates reliable device fabrication, but the actual utilization of this hydrogel has been impeded by its poor fracture toughness, because the required fracture toughness of a hydrogel for an actuator or artificial muscle is 200–500 J m^−2^ [18,19]. Modulus also plays a critical role in achieving practical actuation performance by enhancing fracture toughness; the actuation force increases with increasing modulus [20]. To date, there have been very few reports of hydrogel-base actuators with decent mechanical properties, and the hydrogels that have been reported have not demonstrated pragmatic actuation performance.

In this study, we fabricated a hydrogel-based actuator with a large actuation force by means of enhanced modulus and fracture toughness. Specifically, poly(N-isopropylacrylamide) hydrogel was used as a thermo-sensitive actuator material and covalently crosslinked networks of the PNIPAAm hydrogel were interpenetrated with ionically crosslinking by alginate networks, which helped improve the actuator’s mechanical properties, and its modulus and fracture toughness in particular. These thermally active hydrogels with a tough and large actuation force that we have developed demonstrate practical feasibility for the fabrication of reliable hydrogel-based devices.

## 2. Results and Discussions

To fabricate the hydrogel-based actuator to be tested in this work, poly(N-isopropylacrylamide) hydrogel was used as a thermo-sensitive actuator material. N-isopropylacrylamide has the hydrophobic moiety of the isopropyl group, whereas acrylamide only has the hydrophilic moiety of the amide group. The hydrophobic moiety of the isopropyl group impedes the hydrogen interaction between water molecular and hydrophilic moiety depending on temperature, which results in a thermo-sensitive phase transition associated with the LCST (Lower Critical Solution Temperature) [21]. This phase transition phenomenon facilitates hydrogel-based actuator operation. However, as this pure PNIPAAm hydrogel exhibits soft and brittle properties, it could only exhibit deformation without the application of external force on an object as an actuator. To enhance the mechanical properties, these covalently crosslinked networks of the PNIPAAm hydrogel were interpenetrated with ionic crosslinks by alginates. Alginates are polymeric carbohydrate molecules, and their structures are linearly copolymer, with blocks of mannuronate (M unit) and guluronate (G unit) that are consecutively arranged in M blocks, G blocks, and alternating M and G blocks [15]. The G blocks in one alginate molecule can be ionically crosslinked with various cations. PNIPAAm and alginate-based interpenetrating network hydrogel can be enhanced through the synergy of two mechanisms: crack bridging by a covalently crosslinked network of PNIPAAm by an MBAAm crosslinker, and energy dissipation by unzipping the ionically crosslinked network of alginate by a Ca^2+^ ion crosslinker [15].

**Figure 1:**
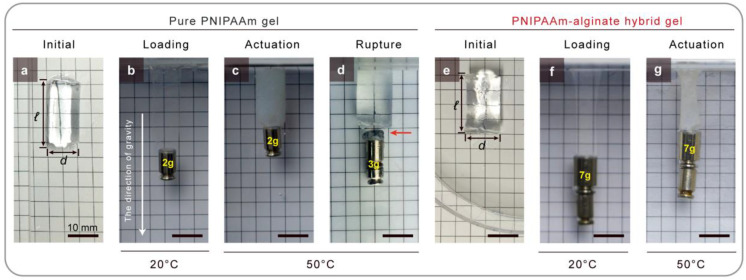
Active materials must be tough. (**a**) Pure PNIPAAm gel was submerged into 20 ℃ water for 1 day and cut into an initial size of *ℓ* = 21 mm with a cylindrical shape (*d* = 10 mm). (**b**) 2 g of brass dead weight was glued to one end of the pure PNIPAAm gel, which was then hung from the ceiling. The gel was stretched by gravity in 20 ℃ water. (**c**) Pure gel with a 2 g brass dead weight was shrunk in 50 ℃ water for 1 day. (**d**) Pure PNIPAAm gels were ruptured by a 3 g brass dead weight in 20 ℃ water (arrow identifies ruptured area in the gel). (**e**) PNIPAAm–alginate hybrid gel fully swollen in 20 ℃ water for a day and cut into a test size of *ℓ* = 20 mm with a cylindrical shape (*d* = 10 mm). (**f**) Hybrid gel capable of sustaining a 7 g brass dead weight in 20 ℃ water. (**g**) Hybrid gel showing thermal actuation against a 7 g brass dead weight in 50 ℃ water. The gel remained intact during actuation.

Figure 1 demonstrates the enhancements in the mechanical properties of the hybrid gels that have been achieved through the formation of interpenetrating networks. As shown in Figure 1a–d, a cylindrically shaped pure PNIPAAm gel was prepared and swollen in 20 °C water for a day. After this swelling period, the pure gel had a length of 20 mm length and a diameter of 10 mm (Figure 1a). To begin, a 2 g brass dead weight was glued onto one end of the pure PNIPAAm gel, at which point the gel was hung from the ceiling. Next, the pure gel was stretched by gravity to 1.4 times its initial length at 20 ℃ (Figure 1b). The temperature of the water was then increased to 50 ℃ to monitor the thermal actuation in 50 ℃ water for a day. After shrinkage in 50 ℃ water for 1 day, the pure gel was able to lift the 2 g brass dead weight approximately 20 mm (Figure 1c). However, because the pure gel was brittle, the gel ruptured during loading when 3 g of dead weight was used, as shown in Figure 1d. In contrast, PNIPAAm–alginate hybrid gel is tough, and it is therefore capable of lifting a heavier dead weight than pure gel. The hybrid gel that was fully swollen in 20 ℃ water for 1 day was prepared with a length of 20 mm and a diameter of 10 mm, and it is shown in its initial state in Figure 1e. The hybrid gel was stretched to 1.5 times its original length with a 7 g brass dead weight (Figure 1f). As shown in Figure 1g, the gel was shrunken to 20 mm from 30 mm length in 50 ℃ water due to the thermal actuation. The hybrid gel was kept intact with 7 g of dead weight during the loading and actuation processes. The results showed that fracture toughness is an essential component to consider for active materials to prevent crack propagation during operation.

**Figure 2:**
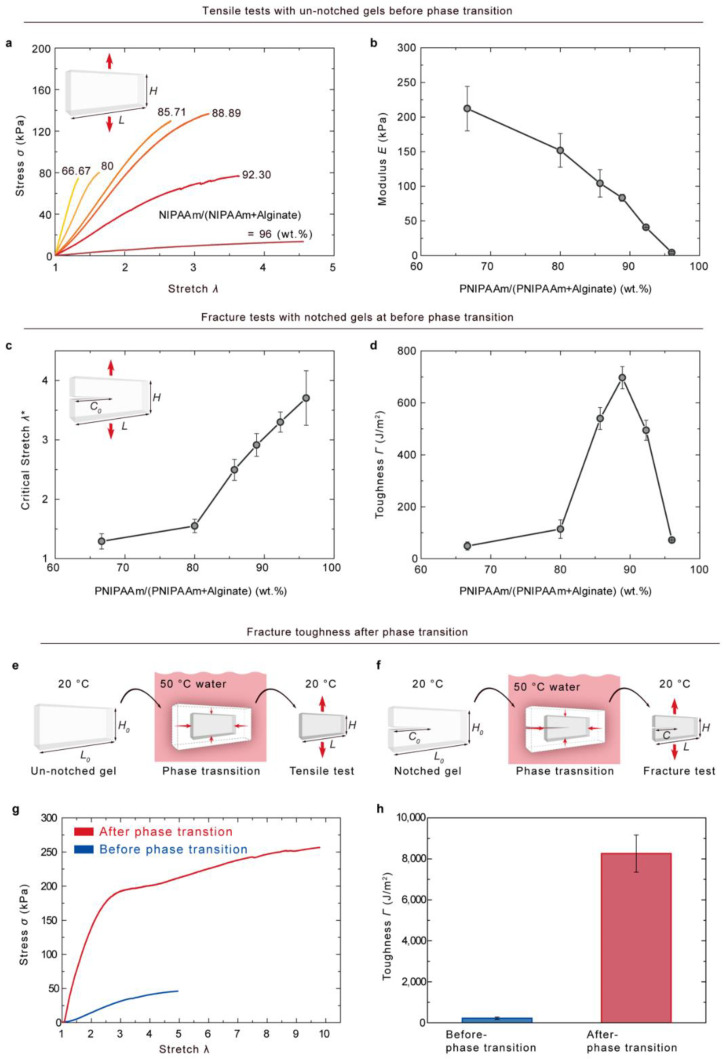
Fracture toughness of PNIPAAm–alginate hybrid gel. (**a**) Un-notched hybrid gel stretched to rupture for a tensile test. Hybrid gels with various weight ratios of PNIPAAm/(PNIPAAm + alginate) were tested. The sample design shown in the inset (*L*/$H_{0}\geq 20$) was used for the tensile tests. The stretch rate was kept constant at $\dot{\lambda }$ = 2 $\min^{-1}$. (**b**) The elastic modulus was calculated from the stress–stretch curves in (**a**) and plotted as a function of the weight ratio of PNIPAAm/(PNIPAAm + alginate). The elastic modulus was increased when the proportion of alginate increased. (**c**) Fracture tests were performed on the notched samples shown in the inset ($C_{0}$ /*L* ≈ 0.5). Onset crack propagation was monitored by a camera during uniaxial stretching. Cracks tended to propagate earlier with increasing amounts of alginate. (**d**) The fracture toughness calculated using Equation (S1) was plotted as a function of the weight ratio of PNIPAAm/(PNIPAAm + alginate). The hybrid gel has a maximum toughness of approximately 700 J/$m^{2}$ at 88.89 wt. %. The weight of the covalent crosslinker, MBAA, was fixed at 0.00167; the weight of the ionic crosslinker, CaSO_4_, was fixed at 0.2327 (water content: 89.59 wt. %). (**e**–**h**), Fracture toughness was also measured after the phase transition. (**e**,**f**) Hybrid gels were fully swollen in 20 ℃ water for a day, then submerged into 50 ℃ water for a day. After the phase transition, un-notched and notched sample gels were subjected to tensile and fracture tests, respectively. (**g**) Un-notched hybrid gel after phase transition shows two times more stretchability and five times more strength than un-notched hybrid gel before the phase transition. As a result, (**h**) the fracture toughness of the hybrid gel after phase transition is 8258 J/m^2^, which is 38 times higher than that of the hybrid gel before phase transition. *H* = 5 mm (C /*L* ≈  0.5). Error bars show standard deviation; *n* ≥3.

PNIPAAm–alginate hybrid gels were synthesized with various weight ratios of PNIPAAm to (PNIPAAm plus alginate) and subjected to tensile tests and fracture tests to measure their mechanical properties. For tensile testing, an un-notched hybrid gel was glued to four stiff polyacrylate clamps, resulting in specimens with initial dimensions of width *L* = 75 mm and thickness *t* = 3 mm, and the distance between the two clamps was *H*_0_ = 5 mm. The specimen was then stretched to rupture using a tensile machine (Instron model 3342). The stretch rate was kept constant at $\dot{\lambda }=2{\;\min}^{-1}$. Figure 2a shows the stress–stretch curves of the hybrid gels with various weight ratios of PNIPAAm to (PNIPAAm plus alginate), as measured by the tensile tests. The stretch, λ, is defined as the distance between the two clamps when the gel is deformed divided by the distance when the gel is at a weight ratio of 96 wt. %, which is close to that of pure PNIPAAm gel; the hybrid gel exhibited the lowest elastic modulus of 4.26 kPa with the highest stretchability of 4.56. With increasing alginate, stretch decreased proportionally, whereas the modulus increased, as shown in Figure 2b. The influence of crosslinker densities on the modulus of the hybrid gel was also investigated, as shown in Figures S1 and S2. The elastic modulus was increased by increasing the CaSO_4_ concentration (Figure S1). However, the concentration of MBAA did not significantly affect the modulus of the hybrid gel (Figure S2). These phenomena may be attributable to the very low stiffness of PNIPAAm networks. Fracture tests were performed with notched samples. The initial notches were made by a razor blade while controlling the initial notch size *C*_o_/*L* ≈ 0.5. The onset of crack propagation was monitored by a camera during an instance of uniaxial stretching. Figure 2c shows the critical stretches of onset crack propagation for different hybrid gels at various weight ratios. When the proportion of alginate was increased, the critical stretch of crack propagation was decreased. The method introduced by Rivlin and Thomas [22] was used to determine the fracture toughness of a gel. The fracture toughness was calculated from the fracture energy equation,

(1) $$\Gamma =\frac{U\left(H_{c}\right)}{Lt}\;J/m^{2}$$

where *L* and *t* are the width and thickness of undeformed samples, respectively, and $U\left(H_{c}\right)$ is the area under the stress–stretch curves from zero to critical stretch ($H_{c}$). Rivlin–Thomas’ methods are explained in further detail in Figure S3. The measured fracture toughness is plotted in Figure 2d as a function of the weight ratios of PNIPAAm/(PNIPAAm + alginate). The fracture toughness of the hybrid gel reached a maximum value of 697.08 J/m^2^ at 88.89 wt. %; this was 58 times higher than that of pure PNIPAAm gel, which is 12 J/m^2^. Further, the measured toughness of the hybrid gel is higher than that of muscles (e.g., horse quadriceps muscle [18] and salmon muscle [19] have fracture toughness values ranging between 200 and 500 J/m^2^). Further, the fracture toughness of the hybrid gel after phase transition was 8258 J/m^2^ (Figure S3). In addition, chemical analysis was performed using FTIR spectroscopy to identify possible changes in the chemical compositions of the hybrid gels (Figure S4). There were no significant changes in the vibrational band for the hybrid gels compared to the pure components.

**Figure 3:**
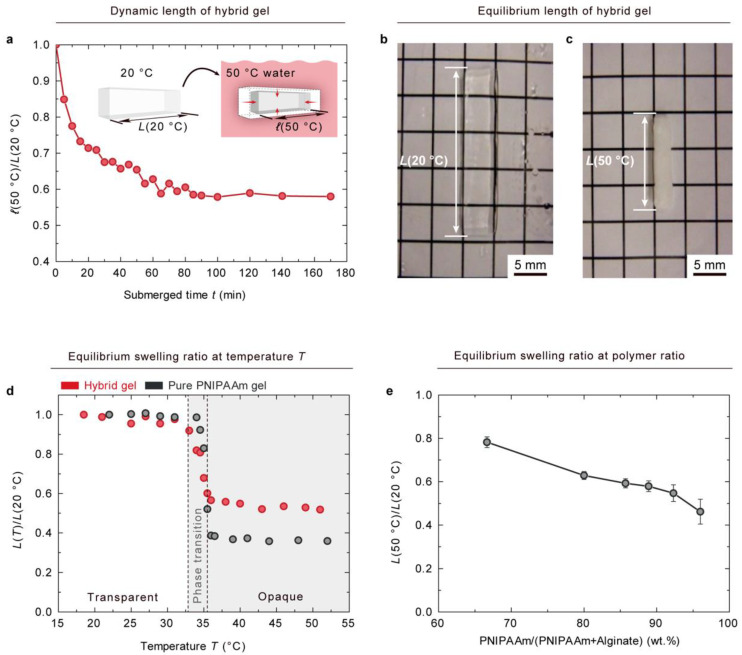
Thermo-sensitive behaviors of PNIPAAm–alginate hybrid gel. (**a**) 20 × 5 × 3 mm^3^ PNIPAAm–alginate hybrid gel, which was fully swollen in 20 ℃ water, was submerged into 50 ℃ water. Dynamic length in 50 ℃ water, *ℓ*(50 ℃ ), was captured as a function of submerged time. The dynamic swelling ratio, *ℓ*(50 ℃ )/*L*(20 ℃ ), of a hybrid gel reached an equilibrium state after 100 min, which is near the theoretical self-diffusion time of 94 min in 50 ℃ water. (**b**) Hybrid gel of PNIPAAm-10.33-0.17/alginate-1.29-22.77 fully swollen in 20 ℃ water. Equilibrium length of the gel in 20 ℃ water is called *L*(20 ℃ ). (**c**) After being submerged in 50 ℃ water, the gel reached another equilibrium state with a length of *L*(50 ℃ ). (**d**) Comparison of equilibrium lengths at temperature *T*, *L*(*T*), of pure gel and hybrid gel. For each data point, the sample was stored under a designated temperature for 4 h to reach an equilibrium state. Both pure and hybrid gels underwent phase transition in a similar temperature range from 32 to 35 ℃. However, the equilibrium swelling ratios, *L*(*T*)/*L*(20 ℃ ), of pure gel and hybrid gel reached steady values of 0.35 and 0.52, respectively. (**e**) Equilibrium swelling ratio of hybrid gel plotted as a function of weight ratios of PNIPAAm/(PNIPAAm + alginate). (Error bars show standard deviation; *n* ≥3).

Next, we investigated the thermo-sensitive behaviors of the prepared hybrid gels. To this end, a hybrid gel of PNIPAAm-10.33-0.17/alginate-1.29-22.77 was fully swollen in 20 ℃ water for a day and cut into a test size with the dimensions of 20 × 5 × 3 mm^3^ by a laser cutter. The gel was then submerged into 50 ℃ water. The dynamic length in 50 ℃ water, *ℓ*(50 ℃), of the hybrid gel was monitored through a camera. Figure 3a plots the dynamic swelling ratio in 50 ℃ water, *ℓ*(50 ℃)/*L*(20 ℃), of the hybrid gel as a function of submerged time, *t*. The dynamic length of the gel was normalized with length at an equilibrium state in 20 ℃ water, *L*(20 ℃). The dynamic swelling ratio of the hybrid gel decreased as a function of submerged time and reached an equilibrium state after 100 min, which is near the theoretical self-diffusion time of 94 min in 50 ℃ water. The theoretical self-diffusion time of thermo-sensitive hydrogel caused by the diffusion of water molecules was calculated using the following diffusion equation,

(2) $$\ell =\sqrt{Dt}$$

where the gel size was 20 × 5 × 3 mm^3^, the water diffusion distance was ℓ = 1.5 mm, and the self-diffusion coefficient of water at 50$\;℃\;\mathrm{was}\;D_{50℃}$ = 3.983·$10^{-9}\;m^{2}/s^{-1}$ [23]. As illustrated in Figure 3b, a transparent hybrid gel of PNIPAAm-10.33-0.17/alginate-1.29-22.77 was fully swollen in 20 ℃ water. The equilibrium length of the gel in 20 ℃ water is called *L*(20 ℃). After being submerged in 50 ℃ water, the hybrid gel reached another equilibrium state at 50 ℃, where it had a length of *L*(50 ℃) and exhibited an opaque appearance (Figure 3c). The equilibrium lengths of the pure gel and hybrid gel at temperature *T*, *L*(*T*), are compared in Figure 3d. To obtain each data point, the sample was stored under the designated temperature for 4 h to reach an equilibrium state. The equilibrium length of the gels at temperature *T*, *L*(*T*), was normalized with the length at an equilibrium state in 20 ℃ water, *L*(20 ℃). *L*(*T*)/*L*(20 ℃) was called the equilibrium swelling ratio. The equilibrium swelling ratio was not changed for either the hybrid or pure gels until 32 ℃. The pure gel and hybrid gel both underwent phase transitions in a similar temperature range from 32 to 35 ℃, which is consistent with the values reported by previous studies [24]. The equilibrium swelling ratio of hybrid gel reached a steady value of 0.52 after the phase transition, which is higher than that of pure gel, 0.35. The volume change in the hybrid gel was lower than that of pure gel. The equilibrium swelling ratios of hybrid gels at a variety of weight ratios are plotted in Figure 3e. The volume change in the hybrid gel was decreased when the proportion of alginate was increased. However, the densities of the ionic and covalent crosslinks in the hybrid gel have little effects on the equilibrium swelling ratio, as shown in Figures S1 and S2.

**Figure 4:**
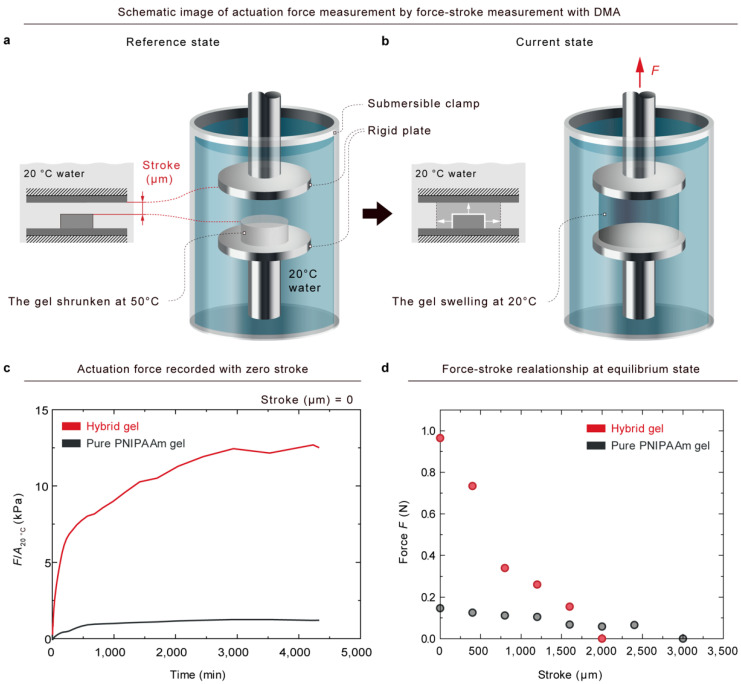
Hybrid gel showing a larger actuation force than pure gel. (**a**) Pure and hybrid gels fully swollen in 20 ℃ water and cut into a disc shape with a diameter of 10 mm. The thicknesses of the pure and hybrid gel were 3.8 mm and 3.4 mm, respectively. The gel was then shrunk in 50 ℃ water for 1 day and placed on a fixed-bottom plate in a submergible clamp kit. An upper plate approached the surface of the gel with a small force of less than 20 µN, and it was lifted from the surface of the gel to create a stroke. The displacements of the upper plate were fixed after giving the stroke between the surface of the hydrogel and the upper plate. The submergible clamp kit was then filled with 20 ℃ water. (**b**) A force, *F*, was generated by a swelling of the hydrogel due to the phase transition. (**c**) The force divided by the cross-sectional area at 20 ℃ was recorded as a function of time submerged with zero stroke. Hybrid gel shows a maximum force 7 times larger than that of pure gel at equilibrium states. (**d**) Force–stroke curves of pure PNIPAAm gel and hybrid gel.

The actuation force was measured by a force–stroke measurement [20], as illustrated in Figure 4a,b. The measurement was conducted under a dynamic mechanical analysis (TA instruments model RSA-G2, New Castle, DE, USA) system with submersible clamp kits. To begin, pure gels and hybrid gels were fully swollen in 20 ℃ water for 1 day. Next, the gels were cut into disc shapes with a diameter of 10 mm by a laser cutter. The thicknesses of the pure and hybrid gel discs at equilibrium state were 3.8 mm and 3.4 mm, respectively. The gel discs were then submerged in 50 ℃ water for 1 day to cause the gel to shrink. The diameters of the pure gel and the hybrid gel after full shrinkage were 5.45 mm and 6.4 mm, respectively. As depicted schematically in Figure 4a, a gel at equilibrium state at 50 ℃ was placed on a fixed-bottom plate in a submergible clamp kit. An upper rigid plate was placed on the surface of the gel with a small force less than 20 μN, and it was lifted from the surface of the gel to make a stroke. The displacements of the upper plate were fixed after a stroke was made between the surface of the hydrogel and the upper plate. A stroke is defined as a displacement between the surfaces of the hydrogel and the upper plate before submergence. The submergible clamp kit was then filled with 20 ℃ water. A force, *F*, was generated by a swelling of the hydrogel due to the phase transition (Figure 4b). As shown in Figure 4c, the force divided by the cross-sectional area of the gel at 20 ℃ was recorded as a function of submerged time with zero stroke. The fully swollen gels generated a maximum stress at an equilibrium state at 20 ℃, where the maximum stresses of the pure PNIPAAm gel (PNIPAAm-11.62-0.17) and hybrid gel (PNIPAAm-10.33-0.17/Alginate-1.29-22.77) were 4 kPa and 26 kPa, respectively. Therefore, the hybrid gel exhibits a maximum force seven times larger than that of pure gel at equilibrium states. The force–stroke relationship is plotted in Figure 4d, where it can be seen that the force–stroke curve of the hybrid gel was steeper than that of the pure gel. According to the results depicted in Figure 2b and Figure 3e, with an increasing proportion of alginate, the modulus increased, whereas the volume change due to swelling decreased. That is, an increase in alginate affects two competing effects for an actuation force: one is that the modulus enhancement effect contributes to an increase in force, and the other is that the swelling reduction effect contributes to a decrease in force. However, the hybrid gel by alginate generated a larger force than pure PNIPAAm gel, as depicted in Figure 4c. The results referenced earlier in this paper demonstrate that the modulus enhancement effect by alginate is more dominant than the swelling reduction effect for an actuation force. Consequently, PNIPAAm–alginate hybrid gel exhibits a larger actuation force than pure PNIPAAm gel in reality, as demonstrated in Figure 1.

**Figure 5:**
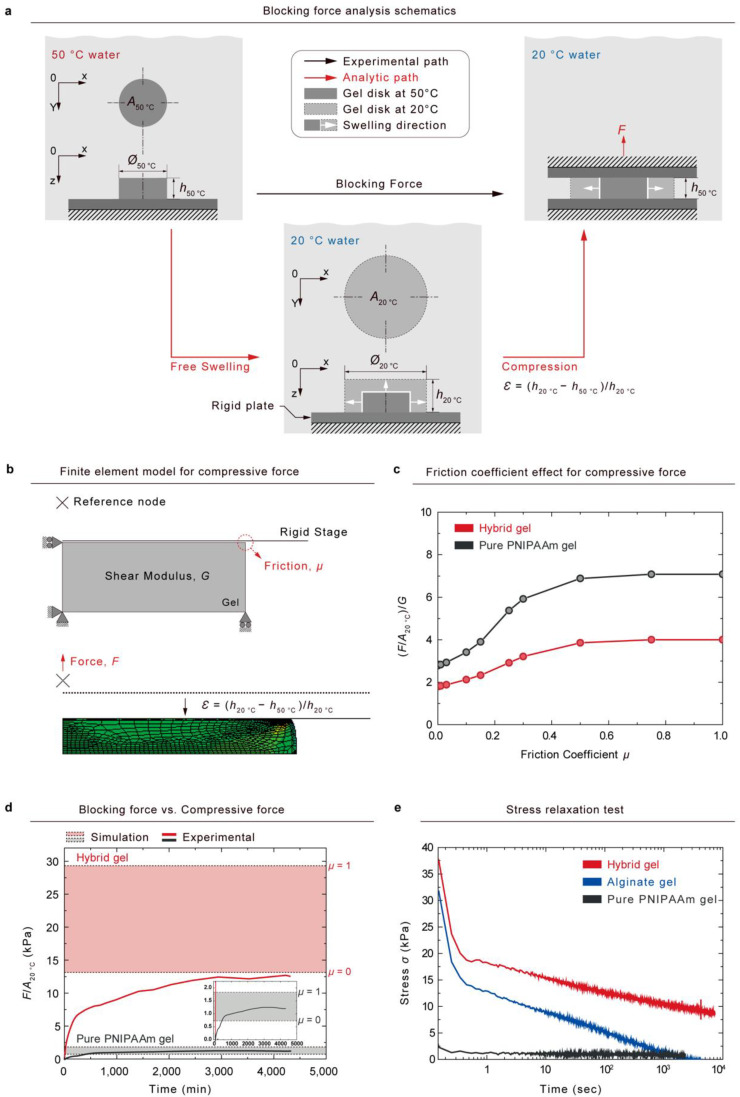
To analyze the blocking force generated by swelling, we examine the compression of the fully swollen gel using the finite element method (FEM). (**a**) We hypothesized that the blocking force generated by the swelling via the phase transition would be equivalent to the compression force applied to the gel at the equilibrium state at 20 ℃. (**b**) Schematic of finite-element model of un-compressed hydrogel with boundary conditions (up). The hyper-elastic model (Neo-Hookean model) simulated the compressed force when strain, ε (*h*_20 °C_− *h*_50 °C_/*h*_20 °C_), was applied (down). (**c**) Simulated–compressed forces plotted as a function of friction coefficient. (**d**) Colored box depicting analytic-compressed force with friction coefficient, ranging from 0 to 1. Line plots corresponding to experimentally measured blocking force. (**e**) Stress–relaxation tests conducted to characterize the viscoelasticity of alginate gel, pure PNIPAAm gel, and hybrid gel.

We hypothesized that the experimentally measured blocking force in 50 ℃ water could be similar to the compression force of fully swollen gel in 20 ℃ water (Figure 5a). The fully shrunken gel disk (Cross sectional area: *A*_50 °C_, Thickness: *h*_50 °C_) generated a blocking force (black color path) that can be measured using the method that we previously described in Figure 4a. When a fully swollen gel disk in 20 ℃ water (cross-sectional area: *A*_20 °C_; thickness: *h*_20 °C_) is compressed to a strain, ε = (*h*_20 °C_−*h*_50 °C_)/*h*_20 °C_ (red color path), the gel will generate a compressive force that we assume to be the same as the experimentally measured blocking force (red color path). We verified the established hypothesis using the finite element method (FEM). Figure 5b depicts a schematic of the Neo-Hookean model of un-compressed gel with boundary conditions. When a strain, ε = (*h*_20 °C_−*h*_50 °C_)/*h*_20 °C_, was applied to the model, compressive forces were varied by the friction coefficient μ between the gel and a stage.

To determine the theoretical maximum and minimum *F/A*_20 °C_ values, we used the ABAQUS finite element code in the simulation of the simple compression of disk-shaped gel. We represent the gel with eight-node axisymmetric solid elements (CAX8R). A rigid body surface was defined to express a compressive stage, and the surface was controlled by a reference node to apply a constant deformation rate. All of the boundary conditions of the finite-element model are detailed in Figure 5b.

We observed that the generated force was influenced by the friction coefficients; when the friction coefficient was increased from 0 to 1, the normalized stress $\frac{F}{A_{20{}^{\circ}C}}\cdot \frac{1}{G}$ for pure PNIPAAm gel and hybrid gel changed by 2.78 to 7.08 and 1.79 to 4.00, respectively (Figure 5c). A comparison of the simulated stress values with the experimentally measured blocking stress is shown in Figure 5d. While the experimental blocking stress of pure PNIPAAm gel was located in the range of the simulated values, the measured blocking stress of the hybrid gel was out of the lower boundary of the simulated values. The viscoelasticity of the gels was analyzed under constant strain using stress–relaxation tests, and the resulting stress was measured over time (Figure 5e). Pure PNIPAAm gel exhibited elastic properties, whereas alginate gel and hybrid gel showed viscoelastic behaviors. The decrease in the blocking stress of the hybrid gel may be attributable to the viscoelastic properties of the hybrid gel. Because hybrid hydrogels contain weak ionic crosslinks, which can relax stress over time, the blocking stress can be decreased below the simulated values.

## 3. Conclusions

In conclusion, we have developed a hydrogel-based actuator with a tough and large actuation force by using poly(N-isopropylacrylamide) hydrogels as thermo-sensitive actuator materials and interpenetrating network structures between the covalently crosslinked poly(N-isopropylacrylamide) and ionically crosslinked alginate. This interpenetrating structure enhanced the mechanical properties of the hybrid gels. By examining these thermally active hydrogels with a tough and large actuation force, we successfully demonstrated their practical feasibility for the fabrication of reliable hydrogel-based devices. The stiffness and fracture energy of the resultant hybrid hydrogel were enhanced by factors of 50 and 14, respectively. In particular, the hybrid gel generated an actuation force up to seven times larger than that of pure gel. These excellent mechanical properties are attributed to the enhanced elastic modulus and energy dissipation in the hybrid gel. Remarkably, the actuating motion of the hybrid hydrogel was available due to a comparable volumetric change with pure PNIPAAm gel. This work demonstrates a simple and diverse fabrication approach that can achieve enhanced mechanical performances for a variety of functional hydrogels, and which extends potential applications of hydrogels in the tissue engineering, artificial muscles, and sensor fields.

## 4. Experimental Section

### 4.1. Materials

N-isopropylacrylamide (NIPAAm; Sigma, 731129, St. Louis, MO, USA) and alginate (FMC Biopolymer, LF 20/40, Philadelphia, PA, USA) were used as the base materials of the network. N,N-methylenebisacrylamide (MBAAm; Sigma, M7279) was used as the cross-linking agent for NIPAAm gel. Ammonium persulfate (APS; Sigma, A9164) and N,N,N’,N’-tetramethylethylenediamine (TEMED; Sigma, T7024) were used as the initiator and accelerator for the redox-radical polymerization, respectively. Calcium sulfate slurry (CaSO_4_•2H_2_O; Sigma, 31221) was used as the ionic crosslinker for the alginate gel. All materials were used as received.

### 4.2. Gel Preparation

The interpenetrating network (IPN) gels were prepared by dissolving alginate and NIPAAm monomer powders in distilled water. The water concentration ($\frac{\mathrm{water}\;\mathrm{wt}.\;\%}{\left(\mathrm{alginate}+\mathrm{NIPAAm}+\mathrm{water}\right)\mathrm{wt}.\;\%}\times 100$) was fixed at 89.59 wt. % throughout the experiments for the IPN, and the polymer ratios were varied by mixing different amounts of alginate and NIPAAm powders. MBAA 0.17 wt. % and APS 0.95 wt. %, with respect to the weight of the NIPAAm monomer, were added as a crosslinker for NIPAAm and an initiator, respectively. Finally, after degassing in a vacuum chamber, calcium sulfate slurry 22.77 wt. % with respect to the weight of the alginate monomer and TEMED 2.5 wt. % with respect to the weight of the NIPAAm monomer were added as the ionic crosslinker for the alginate and accelerator.

Next, the solutions were poured into a glass mold with a 75.0 × 150.0 × 3.0 mm^3^-sized vacancy and covered with a 3 mm-thick transparent glass plate. The gels were cured by an ultraviolet light crosslinker (UVC 500, Hoefer, Holliston, MA, USA) with 8 W power and a 254 nm wavelength at room temperature for 4 h. To avoid heating the gel sample during gelation, the UV crosslinker was kept approximately 10 cm away from the gel sample. Then, before performing the mechanical tests, the gels were left in a humid box for 1 day to stabilize the reactions.

The IPN gels are hereafter referred to as P_1_-x_1_-y_1_/P_2_-x_2_-y_2_, where P_i_, x_i_, and y_i_ (I = 1, 2) are the abbreviated polymer name (i.e., PNIPAAm), the weight concentration of the monomer in wt. % with respect to the weight of water (e.g., $x_{1}=\frac{\mathrm{PNIPAAm}\;\mathrm{wt}.\;\%}{\mathrm{Water}\;\mathrm{wt}.\;\%}\times 100$), and the crosslinker concentration in wt. % with respect to the monomer of the ith network (e.g., $y_{1}=\frac{\mathrm{MBAA}\;\mathrm{wt}.\;\%}{\mathrm{PNIPAAm}\;\mathrm{wt}.\;\%}\times 100$), respectively.


**NIPAAm/(NIPAAm + Alginate) (wt. %)**

**PNIPAAm**

**Alginate**

**x_1_**

**y_2_**

**x_1_**

**y_2_**
66.677.750.173.8722.7780.009.300.172.3222.7785.719.960.171.6622.7788.8910.330.171.2922.7792.3010.730.170.8922.7796.0011.160.170.0522.77100.0011.620.17--

### 4.3. Mechanical Test Preparations

Before the mechanical tests, the surfaces of the hydrogels were dried with N_2_ gas for 1 min to remove water. Four stiff polyacrylate plates were glued with superglue to clamp the gel. In the end, 75.0 (*L*) × 5.0 (*H*_0_) × 3.0 (*t*) mm^3^ test specimens were prepared for the tests. All mechanical tests were performed at room temperature on a tensile machine (Instron model 3342, Norfolk County, MA, USA) with a 500 N-capacity load cell, and the nominal stress and stretch were recorded. The stretch rate was kept constant at $\dot{\lambda }=2{\;\min}^{-1}$.

### 4.4. Thermo-Sensitive Behavior Tests

A 20 × 5 × 3 mm^3^ sample of the hybrid gel was prepared to investigate the dynamic length. Free swelling in the same concentration of calcium ion was conducted to protect against the loss of Ca^2+^ from the gel at 20 ℃ for 1 day. After measuring the length of the fully swollen hydrogel, the gel was submerged in thermostatic 50 ℃ water in a submergible clamp kit. The dynamic length of the hybrid gel was then recorded as a function of submerged time in the range from 20 ℃ to 50 ℃.

Pure PNIPAAm gel and hybrid gel samples were cut into sizes of 20 × 5 × 3 mm^3^ to observe the equilibrium length. Free swelling was examined under the same circumstances via a dynamic length-measurement test. The equilibrium lengths of the hydrogels at each temperature were measured in the range from 20 ℃ to 50 ℃. Each temperature was kept constant for 4 h to ensure that the sample gels reached the equilibrium state, and the equilibrium length of the hydrogels was then measured.

Hybrid gels with various weight ratios of PNIPAAm/(PNIPAAm + alginate) were prepared to record the equilibrium lengths of the hybrid gels and compare the influence of the amount of alginate on equilibrium length. Hybrid gel samples with the dimensions of 20 × 5 × 3 mm^3^ were subjected to an equilibrium length-measurement test with the same test conditions as those previously described.

### 4.5. Constrained Swelling Measurement

Constrained swelling measurement was performed under a dynamic mechanical analysis (TA instruments model RSA-G2) system with submersible clamp kits. Gels were synthesized under 20 ℃ and fully swollen in 20 ℃ water for a day. Disc-shaped gels with a diameter of 10 mm were cut using a laser cutter (Universal Laser System model VLS 3.50). The disc-shaped gels were then fully shrunk in 50 ℃ water for a day. The thicknesses of the samples of pure gel and hybrid gel were 5.45 mm and 6.4 mm, respectively. The gel in equilibrium state was placed at the bottom plate in submergible clamp kits, after which the upper plate was contacted on the surface of the gel sample with a small force of less than 20 µN. The upper plate was fixed after making a stroke. The submergible clamp kit was then filled with 20 ℃ water. The force generated by swelling was recorded as a function of time and stroke.

### 4.6. Viscoelastic Behavior Characterization

Stress–relaxation tests were conducted with an Instron 3342. The gel disks (10 mm in diameter, 3 mm thick) were placed onto the bottom plate. A constant strain of 20% was applied to the gel, while the load was recorded as a function of time. The load normalized by cross sectional area was plotted.

## Data Availability

All data are available in the main text or Supplementary Materials.

## Supplementary Materials

The following supporting information can be downloaded at: https://www.mdpi.com/article/10.3390/gels8050324/s1, Figure S1: Effects of CaSO4 on stress-strain curves; Figure S2: Effects of MBAAm on stress-strain curves; Figure S3: Fracture toughness of gels as measured by the pure shear method introduced by Rivlin and Thomas; Figure S4: FT-IR spectra of alginate, PNIPAAm, and PNIPAAm-alginate hybrid.
