# Supplementary material for: Fracture Toughness and Blocking Force of Temperature-Sensitive PolyNIPAAm and Alginate Hybrid Gels
Source: Gels. 2022 May 23;8(5):324. doi: 10.3390/gels8050324 (PMC9140800; doi:10.3390/gels8050324)
Supplement: Supplementary file 1 [file gels-08-00324-s001.zip › gels-1726063-supplementary.pdf]

gels

MDPI

Supplementary

Fracture Toughness and Blocking Force of Temperature-Sensitive PolyNIPAAm and Alginate Hybrid Gels

Yong-Woo Kim [1,2], Do-Yoon Kim [1,2] and Jeong-Yun Sun [1,2,*]

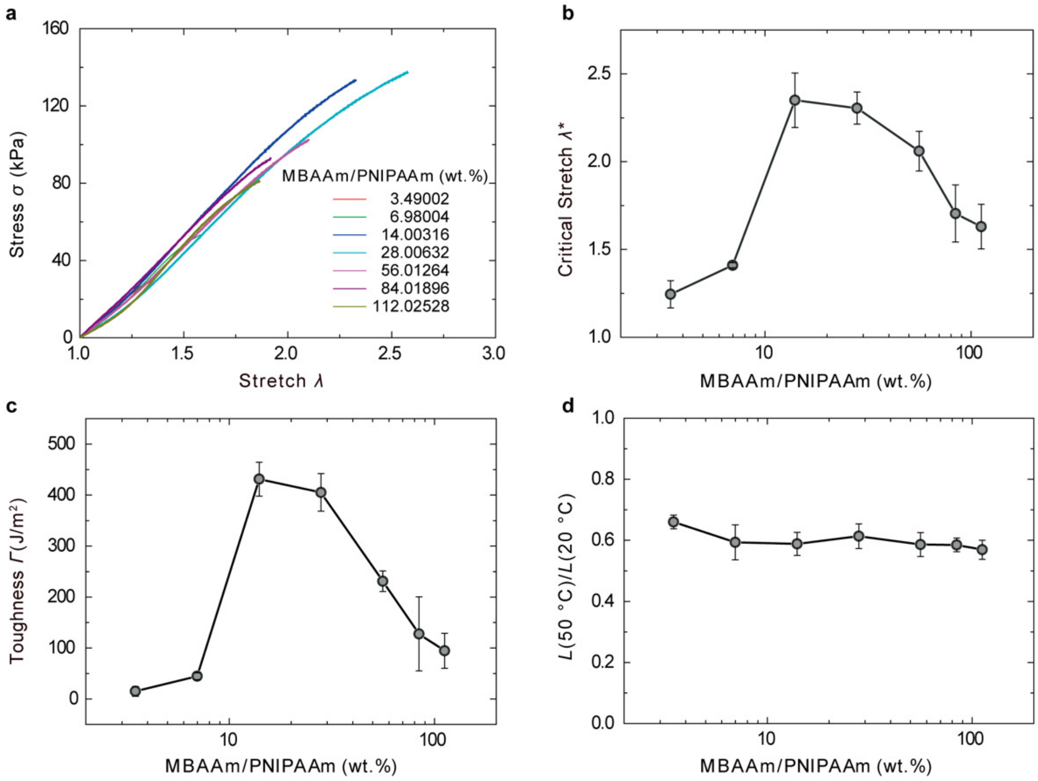

Figure S1. Effects of $CaSO_4$ on stress-strain curves in tensile test (**a**), critical stretch (**b**), toughness (**c**), and temperature sensitivity (**d**). Samples: PNIPAAm-9.96-0.17/Alginate-1.66- y_2 IPN gels with a constant water content of 89.59 wt. %.

The effects of the ionic crosslinks of alginate were investigated for prepared hybrid gels with various concentrations of the crosslinker of $CaSO_4$. With increasing concentration of $CaSO_4$, the density of weak ionic crosslinks for un-notched samples increased, thus causing small-strain elastic modulus and strength to increase as well. However, for the notched sample, the critical stretch turning the notch into a running crack decreased as the concentration of CaSO4 increased. The highest toughness was obtained for an intermediate concentration of $CaSO_4$. As the concentration of $CaSO_4$ increased, the fracture toughness increased because the extent of the interpenetrating network by ionic crosslinks—which dissipates applied energy—increased. At a high concentration of Ca^{2+}, alginate chains are densely crosslinked. Only a small zone around the root of the notch is stressed enough to break the alginate chains, so the fracture energy is low[1]. The concentration of CaSO4 is fully unrelated to temperature sensitivity.

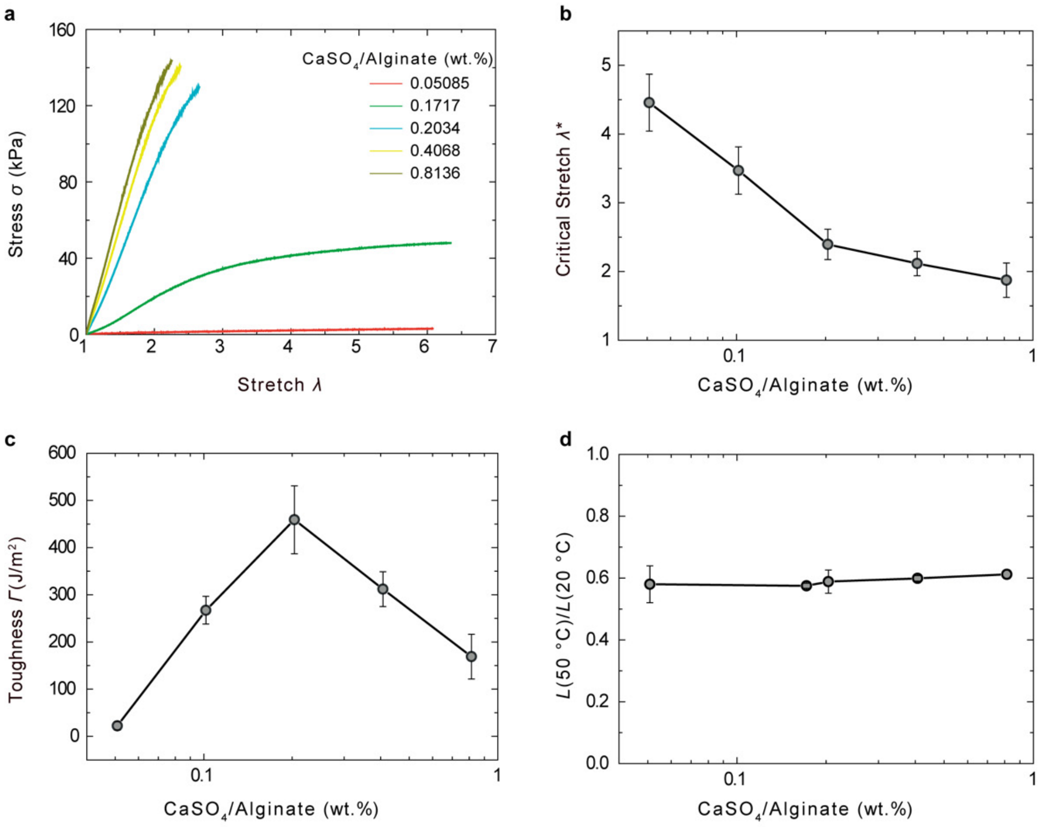

Figure S2. Effects of MBAAm on stress-strain curves in tensile test (**a**), critical stretch (**b**), toughness (**c**), and temperature sensitivity (**d**). Samples: PNIPAAm-9.96-y_1/Alginate-1.66-22.77 IPN gels with a constant water content of 89.59 wt. %.

The effect of the covalent crosslinks of poly(*N*-isopropylacrylamide) was investigated for prepared hybrid gels with various concentrations of the crosslinker of MBAAm. With increasing concentration of MBAAm, the crosslink density of the poly(*N*-isopropylacrylamide) network increased. However, there are no variations of the modulus. The concentration of MBAAm only affects the critical stretches of the notched samples. The highest critical stretches were obtained for an intermediate concentration of MBAAm. As a result, the fracture toughness follows the same trend as critical stretches. As the concentration of the crosslinker MBAAm increased, the fracture toughness increased because the extent of the total network—which sustains an applied load—increased. However, when the covalent crosslink density is too high, each individual poly(*N*-isopropylacrylamide) chain between two crosslinks is short. When the chain breaks, the energy stored in the entire chain is dissipated. Consequently, shorter chains will lead to low fracture energy[1]. The concentration of MBAAm is unrelated to the thermo-sensitive behaviors.

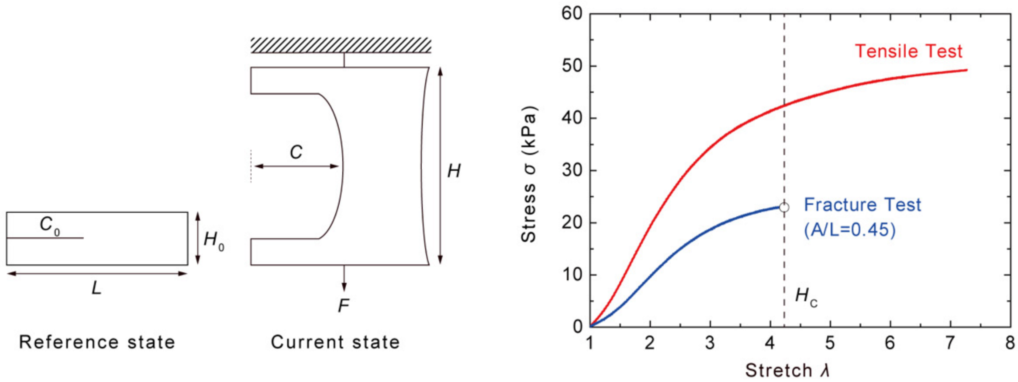

Figure S3. Fracture toughness of gels as measured by the pure shear method introduced by Rivlin and Thomas. Notched and un-notched samples were prepared for tensile tests and fracture tests. The un-notched sample was pulled to measure the force-length curve. The initial samples have a width L = 75 mm and a thickness t = 3 mm, and the distance between the two clamps was H_0 = 5 mm. When the two clamps were pulled to a distance H, the area beneath the force-length curve gave the work done by the applied force, $U(H)$. For a fracture test, the notched sample was prepared using a razor blade to cut a 40 mm-long notch into the gel. The notched sample was pulled, and pictures were taken at a rate of ~30 frames/s to record the critical distance between the clamps, H_c, when the notch turned into a running crack. The fracture energy was calculated by

$$\Gamma = \frac{U(H_c)}{Lt} \quad \mathrm{J/m^2} \qquad \text{(S1)} \qquad (1)$$

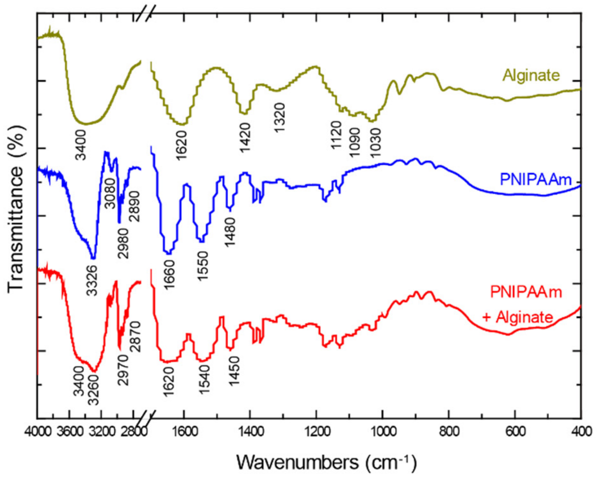

Figure S4. FT-IR spectra of alginate, PNIPAAm, and PNIPAAm-alginate hybrid. For alginate gel, the alcohol O–H stretch and C–O–H bend vibrations are respectively shown at 3400 and 1320 cm^{-1}. Carboxylate salt $–COO^-$ asymmetric and symmetric stretch absorption respectively occur at 1620 and 1420 cm^{-1}. Ether C–O stretch vibrations appears in the range 1120-1030 cm^{-1}. For PNIPAAm gel, secondary amides N–H stretch, C=O stretch, and a combination of C-N stretch and N–H bend vibrations are respectively observed at 3326, 1660, and 1550 cm^{-1}. A Fermi resonance overtone of the 1550 cm^{-1} vibration gives a weak band around 3080 cm^{-1} in secondary amides. Alkane sp^3 C–H stretch, $–CH_2$ bend, and $–CH_3$ bend give vibrational bands between 2980-2890, 1480, and 1390 cm^{-1}, respectively. The spectra of the hybrid gel were characterized by comparing the vibrational bands with the pure components. When the PNIPAAm is mixed with alginate chains, intermolecular

hydrogen bonding weakens the N-H bond, thus shifting the band to a lower frequency, from 3326 to 3260 cm^{-1}.
